# Research on Three-Dimensional Shape Curve Reconstruction Technology for a Scraper Conveyor on an Intelligent Working Face

**DOI:** 10.3390/s23218755

**Published:** 2023-10-27

**Authors:** Fukang Qiao, Xinqiu Fang, Ningning Chen, Minfu Liang, Gang Wu, Fan Zhang

**Affiliations:** 1 School of Mines, China University of Mining and Technology, Xuzhou 221116, China; 2 Research Center of Intelligent Mining, China University of Mining and Technology, Xuzhou 221116, China

**Keywords:** intelligent mining, scraper conveyor, curve reconstruction method, cubic b-spline interpolation, Frenet motion frame, curve prediction interpolation, intelligent working face

## Abstract

“Three straight and two flat” is the inevitable demand when realizing the intelligent mining of a fully mechanized mining face. To address the crucial technical issue of lacking accurate perception of the shape of the scraper conveyor during intelligent coal mining, a three-dimensional curvature sensor involving fiber Bragg grating (FBG) is used as a perceptive tool to conduct curve reconstruction research based on different local motion frames and to reconstruct the shape of the scraper conveyor. Firstly, the formation process of the ‘S’-shaped bending section of the scraper conveyor during the pushing process is determined. Based on the FBG sensing principle, a mathematical model between the variation in the central wavelength and the strain and curvature is established, and the cubic B-spline interpolation method is employed to continuously process the obtained discrete curvature. Secondly, based on differential geometry, a spatial curve reconstruction algorithm based on the Frenet moving frame is derived, and the shape curve prediction interpolation model is built based on a gated recurrent unit (GRU) model, which reduces the impact of the decrease in curve reconstruction accuracy caused by damage to some grating measuring points. Finally, an experimental platform was designed and built, and sensors with curvature radii of 6 m, 7 m, and 8 m were tested. The experimental results showed that the reconstructed curve was essentially consistent with the actual shape, and the absolute error at the end was about 2 mm. The feasibility of this reconstruction algorithm in engineering has been proven, and this is of great significance in achieving shape curve perception and straightness control for scraper conveyors.

## 1. Introduction

With the advent of the fourth scientific and technological revolution, coal mining is currently deeply intertwined with cutting-edge science and technology, such as artificial intelligence and data fusion, and there is movement towards building an intelligent mine with intelligent perception, real-time interconnection, independent decision making, and collaborative control [1]. A scraper conveyor is a crucial component of the transportation equipment used on a coal mining face. It not only works with the shearer to transport coal to the conveyor, but also works with hydraulic support to maintain the stability of the roadway. Therefore, reconstructing the shape curve of a scraper conveyor and determining its straightness through intelligent perception and decision making have become crucial technical challenges in the monitoring of the attitude of stope equipment [2,3,4].

In existing research, the ways of perceiving a scraper conveyor’s shape curve are mainly divided into two categories: One is that of indirect perception methods that invert the shape of the scraper conveyor through the shearer or hydraulic support. In the Landmark project, the Australian Federal Academy of Sciences (CSIRO) proposed a method for detecting the three-dimensional path of a shearer using strapdown inertial navigation technology and inverting the shape of the scraper conveyor for straightening [5]. The principle was to measure the position and attitude of the shearer by using the inertial element installed on it and to calculate the attitude of each middle trough and the shape of the scraper conveyor based on the spatial geometric relationship between the shearer and the scraper conveyor [6,7,8]. The experiment showed that when comparing the measured shape and the actual arranged shape of a scraper conveyor track model, the measurement accuracy could reach ±18 mm [9]. Wang et al. [10,11,12] built a geometric measurement model of a scraper conveyor and developed a measurement instrument with an inertial measurement unit and an axial encoder, and this met the requirements for straightness on a longwall working face. Xie et al. [13] measured the pitch angle of the main body of a shearer and the shape of a scraper conveyor by using a tilt sensor and a strapdown inertial navigation system, and they used an adaptive information fusion algorithm to reflect the real-time shape of the scraper conveyor. Li et al. [14] established a virtual straightening system of a “coal seam + equipment” node, and they proposed a straightening method based on the motion law of a floating connection so that the average straightening error of the scraper conveyor was between 2 mm and 8 mm. Niu et al. [15,16] proposed installing a range finder and sensor on a hydraulic support to complete the straightness and to follow automation control so as to achieve the straightness control of a scraper conveyor.

The other category is that of direct detection methods that directly detect the body shape of a scraper conveyor. Liu et al. [17] applied a vision algorithm to the real-time measurement of the shape of a scraper conveyor so that the straightness of a working face with the conveyor section as a measurement point reached an accuracy of ±50 mm. Lv et al. [18] proposed a three-dimensional straightness monitoring method for a scraper conveyor based on binocular vision and conducted experiments. The experimental results showed that the measurement error was within ±30 mm. Xie et al. [19] created a virtual image of the working state of “three machines” (hydraulic support, shearer, and conveyor) and studied a method of condition monitoring and collaborative dynamic programming for the “three machines”. Zhang et al. [20] used a Kalman filtering algorithm to invert the straightness of a scraper conveyor using digital twin technology, which improved the accuracy of straightness sensing. Although the visual algorithm and digital twin technology achieved certain results in the “three machine” attitude measurement, due to the complex geological conditions of the working face, the optical fiber sensor still has unique advantages in engineering practice [21,22]. Fang et al. [23,24,25,26] developed a three-dimensional curvature sensor involving FBG. Based on the obtained discrete curvature, the spatial positions of the chutes of a scraper were determined through an interpolation algorithm and a curve reconstruction algorithm, and the three-dimensional shape perception of the scraper was realized. In order to solve the rotation problem of the sensor after long-term operation, Song et al. [27] proposed a precision compensation model based on the rotation error angle, and the relative error of the radius of the curvature obtained from the inversion of each discrete point was less than ±6%. However, these studies only used linear interpolation and slope recursion to process the data in the measured area and did not predict the unmeasured area or make it continuous. Therefore, it is necessary to select the appropriate interpolation method and spatial algorithm for transforming curvature information into coordinates in the three-dimensional space so that the real-time perception of the spatial form of a scraper conveyor can be achieved.

To overcome the problem of data loss caused by the destruction of monitoring points due to harsh mining environments, this study used a three-dimensional curvature sensor involving FBG as a sensing tool and derived a spatial curve reconstruction algorithm based on the Frenet motion frame after making the discrete curvature data continuous and combining coordinate transformation and recursion. Subsequently, a real-time shape-sensing system for the scraper conveyor was established using the GRU model. Lastly, an experimental platform for curve reconstruction algorithms was designed and built to assess its feasibility.

## 2. Principle of the Analysis and Perception of a Scraper Conveyor’s Shape Curve

### 2.1. The Analysis of a Scraper Conveyor’s Shape Curve

**Figure 1:**
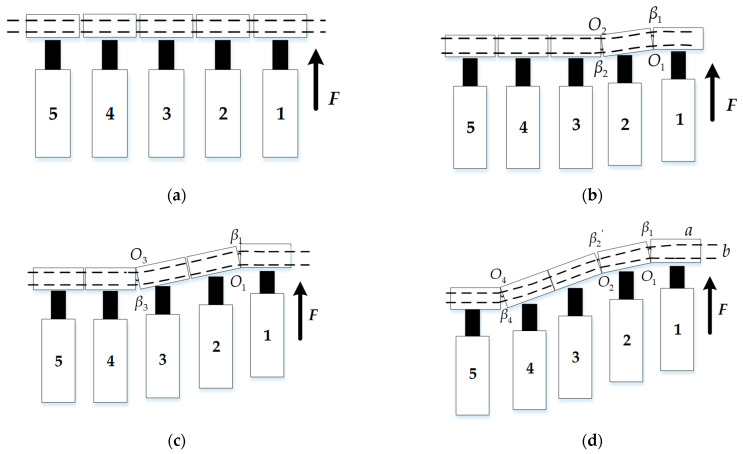
Diagram of the formation process of the ‘S’-shaped bending of a scraper conveyor: (**a**) The first step of ‘S’-shaped bending of scraper conveyor; (**b**) The second step of ‘S’-shaped bending of scraper conveyor; (**c**) The third step of ‘S’-shaped bending of scraper conveyor; (**d**) The fourth step of ‘S’-shaped bending of scraper conveyor.

A scraper conveyor is mainly composed of a head transmission device, scraper chain, scraper chamfer, electrical protection device, chain tensioner, and moving device. The scraper chamfer is made up of several middle chutes connected by dumbbell pins. The presence of dumbbell pins also simplifies the connection of the middle chutes into movable connections that can be rotated in both the horizontal and vertical directions. The middle chutes are connected to the advancing device of a hydraulic support, and its thrust is utilized to power the lateral movement of the scraper conveyor. During the mining process on a fully mechanized mining face, the scraper conveyor is pushed by the hydraulic support; the formation process of ‘S’ -shaped bending is shown in Figure 1.

In the initial state, the scraper conveyor maintains a straight line shape, and hydraulic supports No. 1 to No. 5 correspond to their respective middle chutes, as shown in Figure 1a.

When it starts to move, the hydraulic support provides forward thrust *F* to the corresponding middle chute 1 through the jack, so it protrudes forward. Because the middle chute is connected by pin ears, an angle $\beta_{1}$ with $O_{1}$ as the vertex appears between middle chutes 1 and 2. When middle chute 1 continues to move forward, the thrust is transmitted to middle chute 3, causing it to deflect in the opposite direction. At this time, the angle $\beta_{2}$ with $O_{2}$ as the vertex appears between middle chutes 2 and 3; the shape of the scraper conveyor is shown in Figure 1b.

As the pushing device advances, $\beta_{1}$ and $\beta_{2}$ reach their maximum bending angles in the connecting ear. At this point, middle chutes 1 and 2 can be considered as a whole. The continued thrust causes middle chute 3 to rotate counterclockwise around the hinge point $O_{3}$, generating an angle $\beta_{3}$ between middle chutes 3 and 4. As the angle is formed, angle $\beta_{2}$ gradually decreases, as shown in Figure 1c.

With the ongoing progress of middle chute 1, middle chutes 2 and 3 change from a closed state to a reverse angle ${\beta_{2}}^{\prime }$, and angle $\beta_{3}$ gradually closes. At the same time, angle $\beta_{4}$ appears between middle chutes 4 and 5. Finally, the middle chutes of the scraper conveyor form a symmetrical ‘S’ -shaped bend, which is shown in Figure 1d.

It is assumed that the length of the middle chute of each section of the scraper conveyor is *a*, the width is *b*, and the step distance of each cutting is *S*; the length of the bending section is *L*, and the number of middle chutes is *N*. The expression of the length *L* of the bending section of the scraper conveyor is:

(1) L=aN

The expression of the number of middle chutes in the ‘S’-shaped bending section of the scraper conveyor is:

(2) $$\frac{N}{2}=\frac{1}{\beta }\sec(\cos\frac{\beta }{2}-\frac{(S+b)\sin\frac{\beta }{2}}{a+k})-\frac{1}{2}$$

In the equation, k is the arc length that corresponds to the angle β between the middle chute when bending.

### 2.2. The Principle of the Perception of the Scraper Conveyor’s Shape Curve

#### 2.2.1. The Principle of FBG Strain Sensing

Fiber forms a grating through treatment with ultraviolet light. Due to its restructuring, this grating can filter out incident optical signals. Only an optical signal that matches the specific periodic change can pass through the grating; otherwise, it will be reflected by the grating. The wavelength equation that satisfies the reflection of FBG is:

(3) $$\lambda_{B}=2n_{eff}\Lambda$$

where $\lambda_{B}$ is the central wavelength of the reflected light of the FBG, $n_{eff}$ is the effective refractive index of the fiber core, and Λ is the period of the FBG.

Stress can alter the refractive index of the FBG and change the effective elastic–optic coefficient. When the fiber is only subjected to axial stress, the variation in the central wavelength of the reflected light has the following relationship with the axial strain of the grating:

(4) $$\frac{\Delta \lambda_{B}}{\lambda_{B}}=(1-P_{e})\varepsilon =K_{\varepsilon }\varepsilon$$

where $\Delta \lambda_{B}$ is the center wavelength drift of the reflected light, ε is the axial strain of the grating, $P_{e}$ is the fiber material’s effective elastic–optical coefficient, and $K_{\varepsilon }$ is its strain sensitivity coefficient.

The thermal expansion coefficient and thermo-optic coefficient of an FBG can be affected by temperature changes. When the fiber is only affected by temperature, the variation in the reflected light’s central wavelength has the following effects:

(5) $$\frac{\Delta \lambda_{B}}{\lambda_{B}}=(\alpha +\zeta )\Delta T$$

where α is the coefficient of thermal expansion, ζ is the thermo-optic coefficient, and ΔT is the variation in temperature.

Given the dual effects of ambient temperature and stress, the center wavelength of the FBG’s reflected light can be calculated as follows:

(6) $$\frac{\Delta \lambda_{B}}{\lambda_{B}}=(1-P_{e})\varepsilon +(\alpha +\zeta )\Delta T$$

To reduce the impact of temperature on the measurement outcomes, an unstressed FBG sensor is added for temperature compensation.

#### 2.2.2. The Fundamental Principle of Spatial Curvature Perception

**Figure 2:**
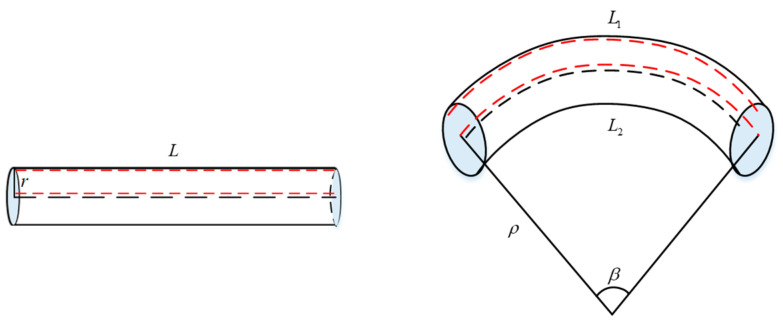
Microelement diagram of the sensor structure.

The three-dimensional curvature sensor involving FBG was made up of multiple sets of FBG shape sensors in series. Each group of FBG shape sensors was encapsulated by a flexible substrate and two orthogonally arranged FBG strings. As depicted in Figure 2, without bending, the sensor remains in a straight state. After applying torque, the radius of the curvature of the bending part is ρ, the corresponding central angle is β, the length of the upper side of the sensor is $L_{1}$, the length of the lower side is $L_{2}$, and the length of the center line remains unchanged. If pure bending occurs, the deformation length of the upper and lower sides is equal, but in reality, tensile deformation occurs when torque is applied, so the different deformations of the upper and lower sides should be taken into account.

After applying strain, the sensor’s upper and lower lengths are:

(7) $$\begin{array}{l} L_{1}=\beta (\rho +r)\\ L_{2}=\beta (\rho -r) \end{array}$$

By combining the equations, the radius of the curvature of the bending part can be calculated as:

(8) $$\rho =\frac{r(L_{1}+L_{2})}{L_{1}-L_{2}}$$

According to the reciprocal relationship between the curvature and radius of curvature, the curvature can be calculated as:

(9) $$k=\frac{1}{\rho }=\frac{L_{1}-L_{2}}{r(L_{1}+L_{2})}$$

When $L_{1}-L_{2}>0$, k>0, the sensor’s bending direction is shown in Figure 2. When $L_{1}-L_{2}<0$, k<0, this indicates that the bending direction is opposite to the direction in the graph.

To analyze the relationship between the curvature and the variation in the center wavelength of the FBG, it is assumed that the sensor has an ideal pure bending, meaning that the upper and lower bending changes are the same. So, the strain on the sensor can be expressed as:

(10) $$\varepsilon =\frac{L_{1}-L}{L}=\frac{L-L_{2}}{L}$$

Substituting it into Equation (9) and combining this with Equation (4), the relationship between the central wavelength variation of the FBG and the curvature is determined:

(11) $$k=\frac{\Delta \lambda_{B}}{(1-P_{e})\lambda_{B}r}$$

Its direction is as follows:

(12) $$\tan\theta =\frac{k_{1}}{k_{2}}$$

Combining Equations (11) and (12), the direction angle θ of the resultant curvature can be expressed as:

(13) $$\theta =\arctan\frac{k_{1}}{k_{2}}=\arctan\frac{\Delta \lambda_{By}\lambda_{Bx}}{\Delta \lambda_{Bx}\lambda_{By}}$$

## 3. The Key Technology of the Shape Curve Reconstruction of a Scraper Conveyor

### 3.1. A Shape Curve Reconstruction Method Based on the Discrete Curvature

**Figure 3:**
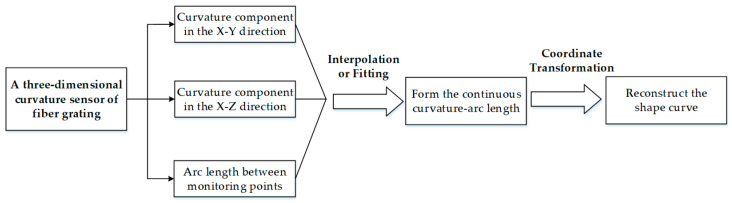
Three-dimensional reconstruction of a scraper conveyor’s shape curve.

The reconstruction of the shape curve with the three-dimensional curvature sensor involving FBG is divided into two steps: The is the interpolation or fitting of the obtained discrete curvature to make it a continuous curvature function; the second is the transformation of the continuous curvature into specific coordinate points in space (Figure 3).

### 3.2. Research on the Discrete Curvature Continuation Method

#### 3.2.1. Analysis of the Interpolation and Fitting Methods

Due to the limited number of measurement points in FBG sensor arrays, the demodulation wavelength range, and the severe mechanical vibration at mining sites, after demodulator sampling, the calculated curvature data are distributed discretely in space, and more curvature information needs to be calculated by using curvature interpolation, fitting, and other methods. There are three commonly used continuous methods. One is the spline interpolation method, which uses polynomials to approximate the function of the curve between data points, resulting in the continuous interpolation of data points. The second is the cubic B-spline interpolation method. Compared to the conventional cubic spline interpolation method, this method has better handling of data point mutations and discontinuities and performs better. The third method is that of least square fitting. By constructing the least square function to match the relationships between data points, a straightforward and accurate curve model can be obtained. A comparative analysis of the advantages and disadvantages of these three methods is presented in Table 1.

#### 3.2.2. Effect Evaluation for the Interpolation and Fitting Methods

**Figure 4:**
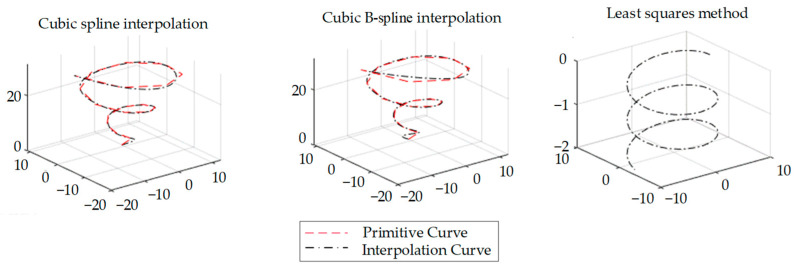
Comparison of the effects of different interpolation methods on conic curves.

First 20 interpolation nodes in the interval [0, 5π] of a standard conic curve were selected, and then the cubic spline interpolation method, the cubic B-spline interpolation method, and the least squares fitting method were used to carry out continuous interpolation; finally, the effects on the continuity of the curve were compared (Figure 4).

The results of the comparison of the different interpolation methods were the following: cubic B-spline interpolation > cubic spline interpolation > least squares fitting method. Therefore, the cubic B-spline interpolation method was chosen in this study to continuously interpolate the discrete curvature measured by the sensor, which provided a rich and continuous basis of curve data to ensure that the subsequent curve reconstruction was accurate.

### 3.3. An Algorithm for Spatial Curve Reconstruction Based on the Frenet Moving Frame

**Figure 5:**
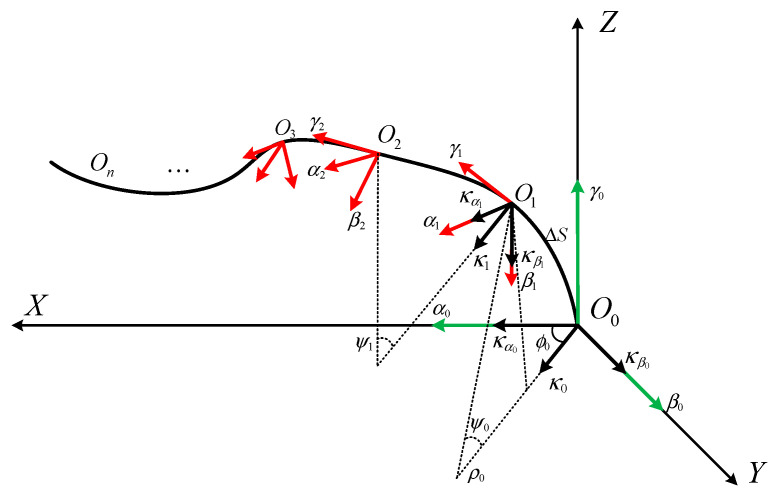
Spatial curve reconstruction diagram.

The principle of the Frenet frame is the establishment of a moving coordinate system for each curve micro-segment at the starting point using the infinitesimal method. The arc length between two monitoring points and the corresponding curvature are used to determine the coordinate value of the next point in the moving coordinate system. Then, the coordinates in the fixed coordinate system are transformed to match a complete three-dimensional curve [28,29]. As shown in Figure 5, the fixed coordinate system is $O_{0}-xoy$, which is recorded as $M^{\left(0\right)}$. The starting point of the coordinate system is at the tail end of the scraper conveyor, and the *x*-axis is defined as the tail pointing towards the head direction. The *y*-axis is the working face’s direction of advance, and the *z*-axis is vertical to the vertical plane. The initial Frenet motion coordinate system $\Gamma^{\left(0\right)}\left\{O_{0};\;\alpha_{0},\;\beta_{0},\;\gamma_{0}\right\}$ is established with point O, and the directions of $\alpha_{0},\;\beta_{0}$ are in line with the directions of the two components of the curve segment’s curvature. The direction of $\gamma_{0}$ is in line with the direction of the tangent vector of the curve segment. Initially, the moving coordinate system coincides with the fixed coordinate system. $O_{1},\;O_{2},\ldots ,O_{n}$ represents the position of the FBG’s monitoring points, and the corresponding motion coordinate system $\Gamma^{\left(1\right)},\;\Gamma^{\left(2\right)},\;\cdots ,\;\Gamma^{\left(n\right)}$ is established with each monitoring point as the origin.

The transformation matrix from the local moving coordinate system $\Gamma^{\left(0\right)}$ at point $O_{0}$ to the fixed coordinate system $M^{\left(0\right)}$ can be expressed as:

(14) $$T_{1}=\left[\begin{array}{cccc} 1 & 0 & 0 & 0\\ 0 & 1 & 0 & 0\\ 0 & 0 & 1 & 0\\ 0 & 0 & 0 & 1 \end{array}\right]$$

The coordinates of the local motion coordinate system $\Gamma^{\left(0\right)}$ are transformed into the coordinates of the local motion coordinate system $\Gamma^{\left(1\right)}$ using a matrix transformation. The process is as follows.

(1) Firstly, the moving coordinate system $\Gamma^{\left(0\right)}$ is rotated around the *z*-axis at an angle of $\phi_{0}$. The rotation transformation matrix can be expressed as:

(15) $$Q_{1}=\left[\begin{array}{cccc} \cos\phi_{0} & -\sin\phi_{0} & 0 & 0\\ \sin\phi_{0} & \cos\phi_{0} & 0 & 0\\ 0 & 0 & 1 & 0\\ 0 & 0 & 0 & 1 \end{array}\right]$$

The rotation angle $\phi_{0}$ is the angle between the measured curvature component $\kappa_{\beta_{0}}$ and the composite curvature $\kappa_{0}$, and the expression is as follows:

(16) $$\left\{\begin{array}{l} \arctan(\frac{\kappa_{\alpha_{n}}}{k_{\beta_{n}}})\;(\kappa_{\alpha_{n}}>0,\kappa_{\beta_{n}}>0)\\ \arctan(\frac{\kappa_{\beta_{n}}}{\kappa_{\alpha_{n}}})+\frac{\pi }{2}\;(\kappa_{\alpha_{n}}>0,\kappa_{\beta_{n}}<0)\\ 0(\kappa_{\alpha_{n}}=0,\kappa_{\beta_{n}}=0)\;\\ \frac{\pi }{2}\;(\kappa_{\alpha_{n}}>0,\kappa_{\beta_{n}}=0)\\ -\frac{\pi }{2}\;(\kappa_{\alpha_{n}}<0,\kappa_{\beta_{n}}=0)\; \end{array}\right.$$

Next, a new moving coordinate system $\left\{O_{0};\;\alpha_{Q_{1}},\;\beta_{Q_{1}},\;\gamma_{Q_{1}}\right\}$ is formed, and the $\beta_{Q_{1}}$-axis direction is the same as the direction of the synthetic curvature $\kappa_{0}$.

(2) The moving coordinate system $\left\{O_{0};\;\alpha_{Q_{1}},\;\beta_{Q_{1}},\;\gamma_{Q_{1}}\right\}$ is rotated around the $\alpha_{Q_{1}}$-axis at an angle of $\psi_{0}$, and the transformation matrix *P*_1_ can be expressed as:

(17) $$P_{1}=\left[\begin{array}{cccc} \cos\psi_{0} & 0 & -\sin\psi_{0} & 0\\ 0 & 1 & 0 & 0\\ \sin\psi_{0} & 0 & \cos\psi_{0} & 0\\ 0 & 0 & 0 & 1 \end{array}\right]$$

The new motion coordinate system $\left\{O_{0};\;\alpha_{P_{1}},\;\beta_{P_{1}},\;\gamma_{P_{1}}\right\}$ is obtained, and the direction of the $\gamma_{P_{1}}$ axis is consistent with the direction of $\gamma_{{}_{1}}$ in the motion coordinate system $\Gamma^{\left(1\right)}$. The rotation angle $\psi_{0}$ can be expressed as:

(18) $$\psi_{0}=\kappa_{0}\Delta s$$

In the equation, the rotation angle $\psi_{0}$ is the central angle of the curve segment $\overset{⏜}{O_{0}O_{1}}$; Δs is the arc length of the curve segment.

(3) The motion coordinate system $\left\{O_{0};\;\alpha_{P_{1}},\;\beta_{P_{1}},\;\gamma_{P_{1}}\right\}$ is rotated around the $\gamma_{p_{1}}$ axis at an angle of $-\phi_{0}$, and the rotation transformation matrix *N*_1_ can be expressed as:

(19) $$N_{1}=\left[\begin{array}{cccc} \cos(-\phi_{0}) & -\sin(-\phi_{0}) & 0 & 0\\ \sin(-\phi_{0}) & \cos(-\phi_{0}) & 0 & 0\\ 0 & 0 & 1 & 0\\ 0 & 0 & 0 & 1 \end{array}\right]$$

A new moving coordinate system, $\left\{O_{0};\;\alpha_{N_{1}},\;\beta_{N_{1}},\;\gamma_{N_{1}}\right\}$, is formed.

(4) The moving coordinate system $\left\{O_{0};\;\alpha_{N_{1}},\;\beta_{N_{1}},\;\gamma_{N_{1}}\right\}$ is moved to point $O_{1}$ so that it aligns with the moving coordinate system $\Gamma^{\left(1\right)}$. The matrix *K*_1_ is expressed as:

(20) $$K_{1}=\left[\begin{array}{cccc} 1 & 0 & 0 & x_{\alpha_{1}}\\ 0 & 1 & 0 & y_{\beta }{}_{1}\\ 0 & 0 & 1 & z_{\gamma_{1}}\\ 0 & 0 & 0 & 1 \end{array}\right]$$

where $x_{\alpha_{1}},\;y_{\beta }{}_{{}_{1}},\;z_{\gamma_{1}}$ are the coordinate values of point *O*_1_ in the fixed coordinate system $M^{\left(0\right)}$, and the size can be expressed as:

(21) $$\left\{\begin{array}{l} x_{\alpha_{1}}=\cos\phi_{0}\times \frac{1-\cos\psi_{0}}{\kappa_{0}}\\ y_{\beta_{1}}=\sin\phi_{0}\times \frac{1-\cos\psi_{0}}{\kappa_{0}}\\ z_{\gamma_{1}}=\frac{\sin\psi_{0}}{\kappa_{0}} \end{array}\right.(\kappa_{0}\neq 0)$$

Thus, the transformation matrix *M*_1_ between the moving coordinate system and the fixed coordinate system can be expressed as:

(22) $$M_{1}=K_{1}N_{1}P_{1}Q_{1}$$

Similarly, the coordinate transformation matrix of the point *O*_n_ in the fixed coordinate system $M^{\left(0\right)}$ can be obtained, and it is expressed as follows:

(23) $$M_{n}=K_{n}N_{n}P_{n}Q_{n}K_{n-1}N_{{}_{n-1}}P_{{}_{n-1}}Q_{{}_{n-1}}\cdots \cdots K_{1}N_{1}P_{1}Q_{1}T_{1}$$

By analogy, points $O_{1},\;O_{2},\ldots ,O_{n}$ can be expressed in a fixed coordinate system $M^{\left(0\right)}$, and then these points are connected to reconstruct the spatial curve.

### 3.4. The Shape Curve Prediction Model for a Scraper Conveyor Based on the GRU

#### 3.4.1. Establishment of the GRU Model

**Figure 6:**
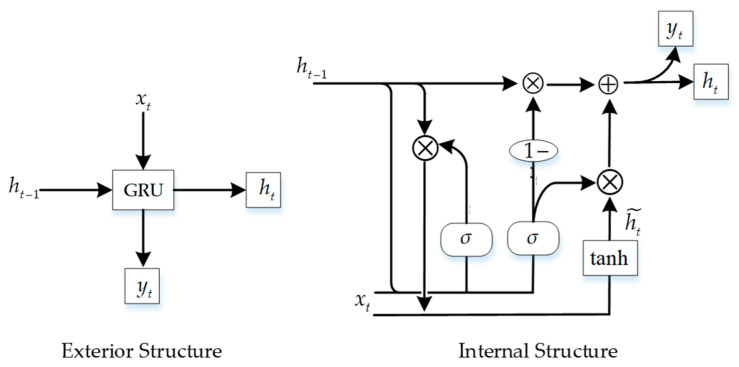
Diagram of the neural network structure of the GRU.

The GRU model creates update reset gates. Through the gating mechanism, past information can be efficiently stored and utilized, and long-term dependencies in the curve data can be captured. The model can also adapt to different data situations through adaptive learning, has a certain fault tolerance for missing data, and can partially restore missing curve information [30]. The neural network structure of the GRU is depicted in Figure 6.

Here, σ, tanh are different activation functions, $r_{t},\;z_{t}$ represent reset and update gates, respectively, and $\tilde{h_{t}}$ is the activation state of the hidden layer at time *t*.

The update gate measures the influence of the output data at the previous moment on the input data at the next moment, and its value range is between 0 and 1. The equation is as follows:

(24) $$z_{t}=\sigma (w_{z}x_{t}+u_{z}h_{t-1})$$

where $x_{t}$ is the input information at time *t*, $h_{t-1}$ is the hidden state of the previous moment, and $w_{i},u_{i}$ are the corresponding weight matrices.

In two nearby locations, the reset gate controls the rate of data forgetting. The value range is between 0 and 1. This equation is as follows:

(25) $$r_{t}=\sigma (w_{r}x_{t}+u_{r}h_{t-1})$$

Once the corresponding states of the update gate and reset gate have been determined, it is necessary to update the hidden layer’s activation state to time *t*:

(26) $${\tilde{h}}_{t}=\tanh\left[w_{c}x_{t}+u_{c}(r_{t}⊗h_{t-1})\right]$$

To conclude, the current hidden state and output information are calculated as follows:

(27) $$\begin{array}{l} h_{t}=(1-z_{t})⊗h_{t-1}+z_{t}⊗{\tilde{h}}_{t}\\ y_{t}=\sigma (w_{o}h_{t}) \end{array}$$

#### 3.4.2. The Prediction and Interpolation Process for the Scraper Conveyor’s Shape Curve

**Figure 7:**
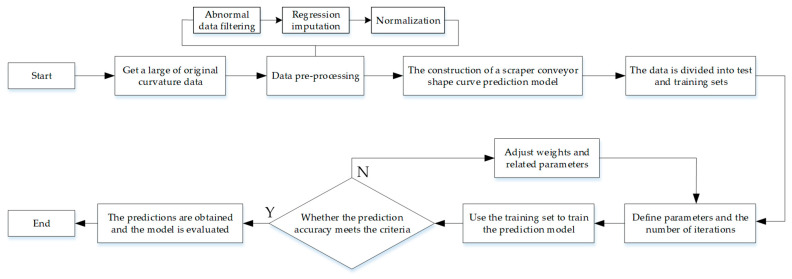
An interpolation flowchart for the prediction of a scraper conveyor’s shape curve.

An illustration of the shape curve prediction interpolation process is shown in Figure 7. Initially, a large number of initial space curve data are obtained and processed. Secondly, the shape curve prediction model for a scraper conveyor is constructed. Finally, the prediction model is trained, and its effect is evaluated.

## 4. The Experimental Testing and Analysis of the Shape Curve Reconstruction Algorithm

### 4.1. Construction of an Experimental Platform for the Reconstruction Algorithm

**Figure 8:**
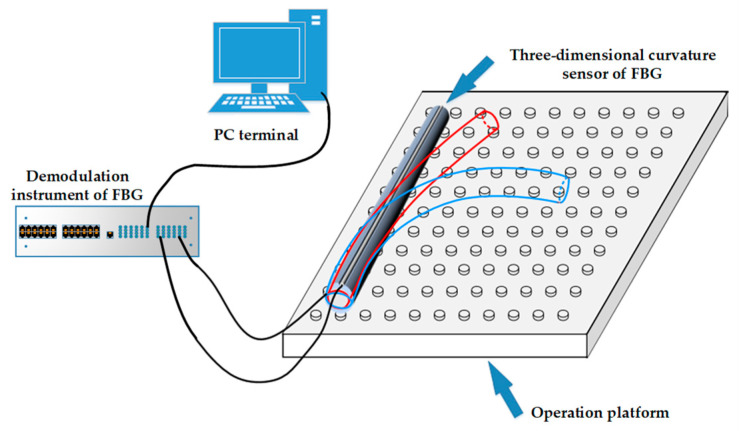
Schematic of the experimental system for verifying the curve reconstruction algorithm.

The experimental system’s hardware was primarily composed of a three-dimensional curvature sensor involving FBG, an SM-125 FBG demodulator instrument, an S178A fiber fusion splicer, and a computer. Two optical fibers were bonded to the grooves while orthogonal to each other on the surface of a polyurethane rod to create a three-dimensional curvature sensor. This was placed on an optical board and bent into various curved radius shapes. The central wavelength shift of each grating point was determined with the demodulator, and the corresponding curvature value was calculated using Equation (11). Subsequently, it was incorporated into the spatial curve reconstruction algorithm for verification, and the corresponding bending shape was displayed on the computer screen. The principle of the curve reconstruction algorithm for experimental systems is illustrated in Figure 8.

In the experiment, two groups of SMF-28e fiber gratings were arranged, and each group was engraved with four grating points. The strain measurement range for each grating point was 0~5000 με, with a maximum tensile force of 100 N. The SM-125 FBG demodulator was used in the experiment, and its main technical parameters are listed in Table 2.

**Figure 9:**
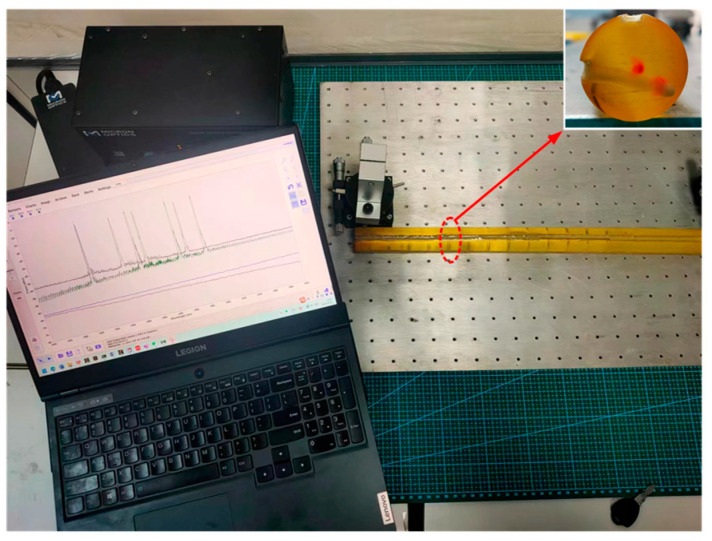
The schematic diagram of a three-dimensional curvature sensor involving FBG.

Due to its strong deformability, the polyurethane rod could be quickly restored after external forces were removed, enabling multiple bending experiments. Therefore, it was used as a packaging substrate for the three-dimensional curvature sensor involving FBG. The grooves, which were orthogonal, were engraved on the surface of the substrate, and an FBG string was embedded within them. The two ends were secured with a pre-tightening device and were uniformly coated with epoxy resin. The sensor was cured at room temperature for 48 h. Figure 9 shows the fabricated three-dimensional curvature sensor involving FBG.

### 4.2. The Experiment on Shape Curve Reconstruction

**Figure 10:**
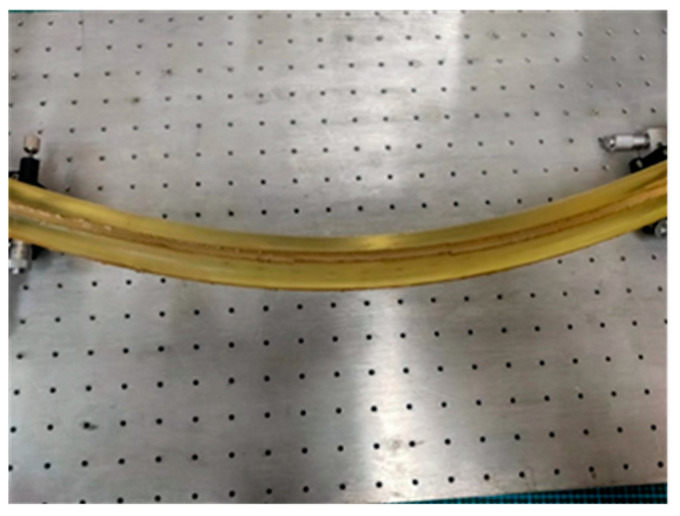
Bending deformation diagram of the sensor.

As shown in Figure 10, the sensor’s end was fixed to the optical experimental platform at one end. By rotating the R-axis platform and the *X*-axis displacement platform at the opposite end, the sensor was bent into arcs with curvature radii of 6 m, 7 m, and 8 m, respectively. After the fiber grating’s wavelength on the display page was stable, the center wavelength drift was measured and substituted into Equation (9) to obtain the curvature data for each grating measuring point. Finally, the curvature was incorporated into the reconstruction algorithm to reconstruct the sensor’s shape curve, and the reconstructed curve was compared with the actual shape to verify the algorithm’s feasibility.

**Figure 11:**
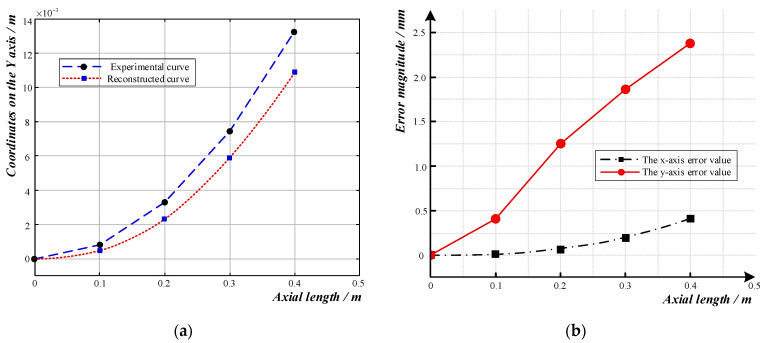
Curve reconstruction and error analysis diagram for a curve with a radius of 6 m: (**a**) Comparison diagram of reconstructed curve and experimental curve; (**b**) Error diagram of reconstructed curve.

The reconstructed curve when the sensor’s bending radius was 6 m is shown in Figure 11a, and the reconstructed shape was essentially the same as the experimental bending shape. As shown in Figure 11b, the error between the reconstructed curve and the actual shape curve in the *x*-axis direction was within 0.5 mm. In the *y*-axis direction, the offset error between the reconstructed curve and the actual curve at 0–0.18 m was within 1 mm; then, the error gradually increased as the axial length increased. At the end, the absolute error reached its maximum, 2.3 mm, while the relative error was 17.79%.

**Figure 12:**
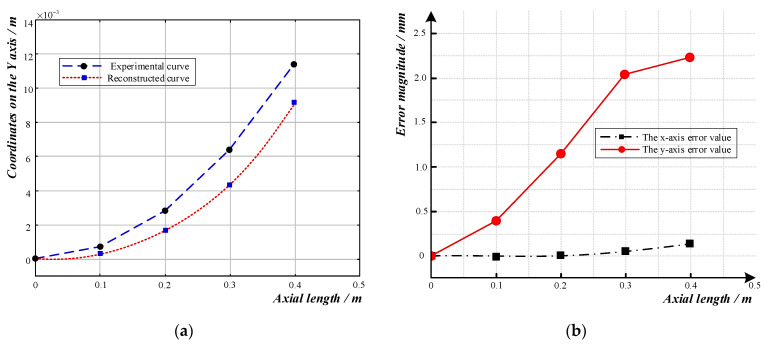
Curve reconstruction and error analysis diagram for a curve with a radius of 7 m: (**a**) Comparison diagram of reconstructed curve and experimental curve; (**b**) Error diagram of reconstructed curve.

As shown in Figure 12a, when the sensor’s curvature radius was 7 m, the reconstructed curve was essentially the same as the actual shape. As shown in Figure 12b, the absolute error at the end of the sensor was 0.1 mm in the *x*-axis direction. In the *y*-axis direction, the error increased as the axial length increased. The data at 0.3 m on the *y*-axis differed from those of the other two sets of bending radii, and they were within the normal fluctuation range. The absolute error at the end was 2.1 mm, while the relative error was 19.61%.

**Figure 13:**
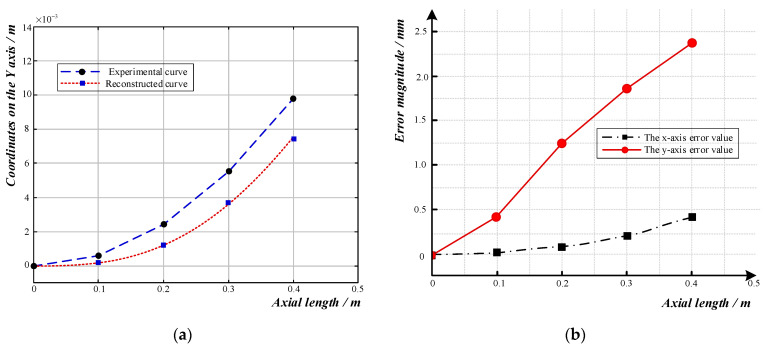
Curve reconstruction and error analysis diagram for a curve with a radius of 8 m: (**a**) Comparison diagram of reconstructed curve and experimental curve; (**b**) Error diagram of reconstructed curve.

When the sensor’s bending curvature radius was 8 m, as shown in Figure 13a, the reconstructed curve was essentially consistent with the actual shape curve. As shown in Figure 13b, the end error values in the *x*-axis and *y*-axis directions were 0.45 mm and 2.2 mm, respectively. In the *y*-axis direction, the error increased as the axial length increased, and the relative error at the end was 19.61%.

From Table 3, it can be seen that the maximum error in the three sets of experiments in the *x*-axis direction was within 0.5 mm, so it was deemed that the curve reconstruction error in the *x*-axis direction was negligible. The three groups of experiments had an absolute error of around 2 mm at the end of the *y*-axis direction, but the relative error increased as the offset at the end of the actual curve decreased. Research and analysis of the mean absolute error and RMSE values of the three parallel experiments showed that the reconstruction algorithm is feasible and has high reconstruction accuracy in practical applications.

## 5. Conclusions

1. A spatial curvature perception theory for a three-dimensional curvature sensor involving FBG is proposed, and a mathematical model that included the central wavelength variation of the FBG, strain, and spatial curvature was established, which established a foundation for the reconstruction of the shape curve of a scraper conveyor.
2. The cubic B-spline interpolation method produced a better result when used to continuously interpolate a standard conic curve. The spatial curve reconstruction algorithm based on the Frenet moving frame was able to reconstruct the curve in a fixed coordinate system.
3. The shape curve of a scraper conveyor was accurately reconstructed using the prediction model, which was based on the fundamental principle of the GRU model, even if some grating measuring points were damaged over time.
4. Indoor experiments showed that the reconstruction curves obtained with the reconstruction algorithm were essentially consistent with the actual shape when the curvature radius of the three-dimensional sensor involving fiber grating was 6 m, 7 m, or 8 m, which proved that the reconstruction algorithm is feasible in practical application.
5. During the ‘S’-shaped bending experiment, due to the excessive bending strain on the substrate, the FBG was pulled off. Therefore, based on the principle of symmetry, half of the ‘S’-shaped data were selected for the bending experiment, thus reducing the reliability. There are limitations, and further research is needed to improve this experiment.

## Figures and Tables

**Table 1:** Comparative analysis of discrete curvature continuation methods.

Methods	Advantages	Disadvantages
Third-order spline interpolation method	1. This method can produce smooth interpolation curves with good shape-fitting capabilities; 2. This method can reduce error in high-data-point-density cases.	1. This method requires a lot of calculation, and the time increases significantly for large-scale datasets; 2. In the event of jumps or mutations between data points, the interpolation may not be accurate.
Cubic B-spline interpolation method	1. This method is better at handling data mutation and discontinuity and making the interpolation results more accurate; 2. In the case of high data point density, this method’s error is small.	1. The calculation time for this method is significant and increases significantly for large-scale datasets; 2. The theory behind it is complex, and the algorithm requires optimization and improvement in real-world applications.
The least square fitting method	1. Models constructed using this method are simple and easy to understand and implement; 2. This method can handle linear relationships well and has high fitting accuracy for data with less noise.	1. This method is not effective in dealing with nonlinear relationships and may result in a loss of fit accuracy for data with high noise; 2. This method can only handle a limited number of independent variables and has limitations when dealing with high-dimensional data.

**Table 2:** Technical parameters of the SM-125 FBG demodulator.

Technology Index	Parameter
Demodulation wavelength range	1510 nm–1590 nm
Strain resolution	1 pm
Channel number	4 channels (the number of channels can increase to 16)

**Table 3:** Error statistics for the reconstructed curves.

Radius of Curvature /m	Absolute Error of *x*-Axis End/mm	Absolute Error of *y*-Axis End/mm	Relative Error of *y*-Axis End	Mean Absolute Error	RMSE
6	0.45	2.3	17.79%	$1.027\times 10^{-3}$	$1.3284\times 10^{-3}$
7	0.1	2.1	19.61%	$1.160\times 10^{-3}$	$1.6249\times 10^{-3}$
8	0.45	2.2	22.99%	$1.449\times 10^{-3}$	$1.6108\times 10^{-3}$

## Data Availability

This study did not report any data.
